# Thrombospondin-1/CD47 signaling modulates transmembrane cation conductance, survival, and deformability of human red blood cells

**DOI:** 10.1186/s12964-020-00651-5

**Published:** 2020-09-18

**Authors:** Rosi Bissinger, Polina Petkova-Kirova, Olga Mykhailova, Per-Arne Oldenborg, Elena Novikova, David A. Donkor, Thomas Dietz, Abdulla Al Mamun Bhuyan, William P. Sheffield, Marijke Grau, Ferruh Artunc, Lars Kaestner, Jason P. Acker, Syed M. Qadri

**Affiliations:** 1 Department of Internal Medicine, Division of Endocrinology, Diabetology, and Nephrology, Universitätsklinikum Tübingen, Tübingen, Germany; 2 Institute of Neurobiology, Bulgarian Academy of Sciences, Sofia, Bulgaria; 3 Centre for Innovation, Canadian Blood Services, Edmonton, AB Canada; 4 Department of Laboratory Medicine and Pathology, University of Alberta, Edmonton, AB Canada; 5 Department of Integrative Medical Biology, Umeå University, Umeå, Sweden; 6 Centre for Innovation, Canadian Blood Services, Hamilton, ON Canada; 7 Department of Pathology and Molecular Medicine, McMaster University, Hamilton, ON Canada; 8 Institute of Molecular and Cellular Sports Medicine, German Sport University of Cologne, Köln, Germany; 9 Department of Physiology, Eberhard-Karls University, Tübingen, Germany; 10 Institute of Diabetes Research and Metabolic Diseases of the Helmholtz Center Munich at Eberhard-Karls University, Tübingen, Germany; 11 German Center for Diabetes Research (DZD), Eberhard-Karls University, Tübingen, Germany; 12 Theoretical Medicine and Biosciences, Saarland University, Homburg, Germany; 13 Experimental Physics, Saarland University, Saarbruecken, Germany; 14 Faculty of Health Sciences, Ontario Tech University, Oshawa, ON Canada

**Keywords:** Thrombospondin-1, CD47, Red blood cells, Calcium, Cation channels, Deformability

## Abstract

**Background:**

Thrombospondin-1 (TSP-1), a Ca^2+^-binding trimeric glycoprotein secreted by multiple cell types, has been implicated in the pathophysiology of several clinical conditions. Signaling involving TSP-1, through its cognate receptor CD47, orchestrates a wide array of cellular functions including cytoskeletal organization, migration, cell-cell interaction, cell proliferation, autophagy, and apoptosis. In the present study, we investigated the impact of TSP-1/CD47 signaling on Ca^2+^ dynamics, survival, and deformability of human red blood cells (RBCs).

**Methods:**

Whole-cell patch-clamp was employed to examine transmembrane cation conductance. RBC intracellular Ca^2+^ levels and multiple indices of RBC cell death were determined using cytofluorometry analysis. RBC morphology and microvesiculation were examined using imaging flow cytometry. RBC deformability was measured using laser-assisted optical rotational cell analyzer.

**Results:**

Exposure of RBCs to recombinant human TSP-1 significantly increased RBC intracellular Ca^2+^ levels. As judged by electrophysiology experiments, TSP-1 treatment elicited an amiloride-sensitive inward current alluding to a possible Ca^2+^ influx via non-selective cation channels. Exogenous TSP-1 promoted microparticle shedding as well as enhancing Ca^2+^- and nitric oxide-mediated RBC cell death. Monoclonal (mouse IgG1) antibody-mediated CD47 ligation using 1F7 recapitulated the cell death-inducing effects of TSP-1. Furthermore, TSP-1 treatment altered RBC cell shape and stiffness (maximum elongation index).

**Conclusions:**

Taken together, our data unravel a new role for TSP-1/CD47 signaling in mediating Ca^2+^ influx into RBCs, a mechanism potentially contributing to their dysfunction in a variety of systemic diseases.

Video abstract

## Background

Thrombospondin-1 (TSP-1), a multimodular Ca^2+^-binding trimeric matricellular glycoprotein, is secreted by a wide array of cells such as platelets, fibroblasts, macrophages, dendritic cells, vascular smooth muscle cells, keratinocytes, epithelial cells, endothelial cells, and several cancer cells [1–3]. Alterations in TSP-1 expression levels have been implicated in the pathophysiology of several clinical conditions including diabetes, cancer, renal failure, and cardiovascular diseases [4–6]. TSP-1 possesses interacting domains for a variety of proteins, which relay signals regulating a diverse range of cellular functions such as cytoskeletal organization, migration, cell-cell interaction, cell proliferation, autophagy, and apoptosis [2, 7, 8]. Mechanisms of TSP-1-induced apoptosis, widely characterized in endothelial and cancer cells, have been reported to involve caspases, NF-κB, Bax, Bcl-2, nitric oxide (NO), as well as p38 and c-Jun N-terminal kinases [9, 10]. TSP-1-induced apoptosis has been largely ascribed to its binding with tumor necrosis factor receptor-1, scavenger receptor CD36, and the integrin-associated protein CD47 [10]. CD47, a ubiquitously expressed glycosylated cell surface protein, regulates cell activation or survival, depending on the physiological context [11, 12]. TSP-1 signaling via its cognate receptor CD47 has further been implicated in orchestrating cytoplasmic Ca^2+^ dynamics and, thus, influencing various physiological functions [12].

In mature red blood cells (RBCs), CD47 is associated with different membrane proteins forming linkages with both cytoskeletal and non-cytoskeletal cellular components [11]. CD47 is pivotal in inhibiting RBC phagocytosis via binding to signal regulatory protein α (SIRPα) on macrophages, which counteracts phagocytosis of non-opsonized as well as IgG or complement-opsonized RBCs [13, 14]. A decline of cell surface CD47 expression during RBC aging in vivo is believed to promote the clearance of senescent RBCs [15]. Furthermore, microparticle release during RBC storage has been reported to favor CD47 loss during storage of RBCs for transfusion [16]. Beyond its significance in RBC aging, CD47 mediates the interaction of fibrinogen with the RBC membrane [17], and may, therefore, contribute to RBC hyperaggregation and altered hemorheology in inflammatory conditions [18, 19].

Similar to apoptosis of nucleated cells [10], ligation of CD47 with monoclonal antibodies, TSP-1, or its derivative peptides has been shown to trigger phosphatidylserine (PS) exposure on RBC cell surface with a concomitant loss of their viability [20]. The mechanisms underlying this phenomenon in RBCs, however, remain elusive. PS externalization, a cardinal morphologic sign of cell death (sometimes also referred to as eryptosis), is stimulated by activation of Ca^2+^-sensitive scramblases [21–23]. Influx of extracellular Ca^2+^ into the RBC cytoplasm is mediated by voltage-gated and voltage-independent non-selective cation channels (NSCC [24, 25]), which are activated by pathophysiologic cell stressors such as hyperthermia, oxidative stress, extracellular hyperosmolality, and starvation [21, 26]. Supraphysiologic Ca^2+^ overload in RBCs induces metabolic reprogramming [27], and activation of multiple enzymes [21], thereby eliciting cellular dysfunction and death. PS-exposing RBCs are rapidly cleared from the circulation and catabolized by macrophages of the reticuloendothelial system in the spleen and liver [21, 28].

In the present study, using cytofluorometric and electrophysiological approaches we examined the influence of CD47-dependent signaling, evoked by exogenous TSP-1 or antibody-mediated CD47 ligation, on Ca^2+^ dynamics in human RBCs. We further studied the effect of TSP-1 exposure on multiple parameters of RBC deformability and cell death.

## Methods

### RBCs and reagents

Leuko-depleted RBC concentrates were provided by Canadian Blood Services (CBS) Network Centre for Applied Development (netCAD, Vancouver, BC, Canada) after prior approval from the CBS Research Ethics Board (#2015.022). For some experiments, these concentrates were provided by the blood bank of the University of Tübingen (#184/2003 V), Germany, or by the blood bank of Norrlands University Hospital, Umeå, Sweden. Donor RBCs, drawn from refrigerated blood bags containing SAG-M additive solution, were washed twice in PBS (1000×g for 10 min) and subsequently incubated in vitro (1% hematocrit unless indicated otherwise) at 37 °C in Ringer’s solution (pH 7.4) containing 125 mM NaCl, 5 mM KCl, 1 mM MgSO_4_, 32 mM HEPES, 5 mM glucose, and 1 mM CaCl_2_. Sample sizes (number of RBC units; n) for control and treatment groups used in individual experiments are indicated in the figure legends. Where indicated, RBCs were incubated with recombinant human thrombospondin-1 (1–50 μg/mL; R&D Systems, Minneapolis, MN, USA) or with anti-human CD47 mAb 1F7 (mouse IgG1), which was purified from hybridoma supernatants [29–31]. In some experiments, RBCs were treated with sodium nitroprusside (Sigma Aldrich, Taufkirchen, Germany) or amiloride (Sigma Aldrich), as described in the figure legends.

### Flow cytometry

Multiple indices of RBC cell death were analyzed using flow cytometry. After incubation under the respective experimental conditions, RBCs were washed once and phospholipid scrambling, intracellular Ca^2+^, and the generation of reactive oxygen species were examined using annexin V-FITC (1: 200 dilution; ImmunoTools, Friesoythe, Germany), Fluo-3/AM (5 μM, Biotium, Hayward, USA), and 2′,7′-dichlorodihydrofluorescein diacetate (10 μM, Sigma) staining, respectively [32]. Ceramide abundance was determined using a previously described monoclonal antibody-based assay with a primary anti-ceramide antibody (1:50 dilution; clone MID15B4; Alexis, Grünberg, Germany) and a fluorescent secondary antibody (1:500 dilution; FITC-conjugated goat anti-mouse IgG/IgM; BD, San Jose, CA, USA) [32]. Data were analyzed using FlowJo software (FlowJo LLC, Ashland, OR, USA). Fluorescence parameters in all samples were analyzed at an excitation wavelength of 488 nm and an emission wavelength of 530 nm.

RBC morphology and microparticle (MP) generation were examined simultaneously using high-throughput imaging flow cytometry, which enables qualitative phenotypic screening of both parent RBCs and MPs, and thereby circumvents the limitations of conventional flow cytometry [33]. Using differences in scattering intensities, RBCs, ghosts, and MPs were characterized and their concentrations (objects) were determined by extrapolating the area of each subpopulation for a given sample [33, 34]. To tackle inter-sample variability in RBC and MP counts in the control and treated groups, the relative concentration of MPs in the sample, calculated as a ratio of RBC-derived (CD47^+^ CD235a^+^) MPs to total RBC, was determined. For RBC morphology index (MI) assessment, a sequentially-numbered set of individually captured RBC brightfield images (170 ± 7 images per sample) was manually assigned by a human operator to six morphology subclasses: smooth discs (SDCs), crenated discs (CDCs), crenated discoids (CDDs), crenated spheroids (CSDs), crenated spheres (CSEs), smooth spheres (SSEs), and multiplied by fractional weights [35]:

$$ \mathrm{MI},\%=\left(\left(\mathrm{SDCs}\times 1.0\right)+\left(\mathrm{CDCs}\times 0.8\right)+\left(\mathrm{CDDs}\times 0.6\right)+\left(\mathrm{CSDs}\times 0.4\right)+\left(\mathrm{CSEs}\times 0.2\right)+\left(\mathrm{SSEs}\times 0\right)\ast 100\right)/\left(\mathrm{SDCs}+\mathrm{CDCs}+\mathrm{CDDs}+\mathrm{CSDs}+\mathrm{CSEs}+\mathrm{SSEs}\right). $$

After a 48-h incubation with 50 μg/mL TSP-1 (at 40% hematocrit) in Ringer’s solution, 75-μL of the cell suspension was washed once and adjusted to 100 μL with Ringer’s solution. For qualitative analyses, the control and TSP-1-treated RBC samples containing all three subpopulations were then incubated at room temperature (30 min; under protection from light) with 5 μL of CD47 PerCP-Cy™5.5 (BD Pharmingen™, clone B6H12) and 2.5 μL of 20 μg/mL CD235a BV510 (BD Pharmingen™, clone GA-R2 (HIR2)). The samples were then examined on an ImageStreamX MkII instrument (ISX; Amnis/MilliporeSigma) equipped with 4 lasers (405 nm, 488 nm, 642 nm, 785 nm (SSC)) and 3 objectives (20×, 40×, and 60×). All data was acquired at 60× magnification, 7 μm core size and low flow rate. CD47 PerCP-Cy5.5 signals were collected in channel 5 (642–745 nm filter) and CD235a BV510 signals in channel 8 (505–570 nm filter). Channels 1 (420–480 nm filter) and 9 (570–595 nm filter) were used as Bright Field channels (BF1, BF2) and channel 12 (745–800 nm filter) for SSC detection (Dark Field (DF) Scattering intensity). Data analyses was performed using Amnis IDEAS software (version 6.2).

### Electrophysiology

Patch-clamp measurements were performed with a NPC-16 Patchliner (Nanion Technologies, Munich, Germany). The internal and external solutions were as follows: KCl 70 mM, KF 70 mM, NaCl 10 mM, HEPES 10 mM, MgATP 2 mM, EGTA 3 mM, and CaCl_2_ 1.2 mM to give 120 nM free [Ca^2+^]_i_, pH = 7.2 adjusted with KOH (internal) and NaCl 140 mM, KCl 4 mM, MgCl_2_ 5 mM, D-glucose 5 mM, HEPES 10 mM, CaCl_2_ 2 mM, pH = 7.3 adjusted with NaOH (external). In these solutions, the resistance of the chips was between 5 and 8 MΩ. Gigaseal formation was facilitated using a seal enhancing solution as recommended by the Patchliner manufacturer and containing: NaCl 80 mM, KCl 3 mM, MgCl_2_ 10 mM, CaCl_2_ 35 mM, HEPES 10 mM, pH = 7.3 adjusted with NaOH. Whole-cell configuration was achieved by negative pressure suction pulses between − 45 mbar and − 150 mbar and its formation judged by the appearance of sharp capacitive transients. Whole-cell patch-clamp recordings were conducted at room temperature using voltage steps from − 100 mV to 80 mV for 500 ms in 20 mV increments at 5 s intervals, the holding potential being set at − 30 mV. Whole-cell currents were assessed before (control) and after adding 50 μg/mL TSP-1. To reduce inter-cell variability, data are expressed as normalized current, which is the ratio of the current under specified experimental conditions, i.e. before and in the presence of 50 μg/mL at the membrane potentials used in the protocol, to the current at + 80 mV determined 30–60 s before starting the control measurement.

### RBC deformability measurement

RBCs were incubated (40% hematocrit) for 48 h at 37 °C. After incubation time, 250 μL of control or TSP-1-treated RBCs were washed once with Ringer’s solution. Ten μL of the aliquot was transferred into 1 mL viscous PVP (polyvinylpyrrolidone; RR Mechatronics, The Netherlands) for RBC deformability measurements. RBC deformability was measured using the laser assisted optical rotational red cell analyzer (LORRCA; RR Mechatronics, The Netherlands). The two parameters used to describe RBC deformability are EI_max_ and K_EI_. The EI_max_ is defined as the maximum elongation index predicted at an infinite shear stress. The K_EI_ is the shear stress required to elongate to half the EI_max_. These parameters are obtained using an Eadie-Hofstee linearization, which plots the measured EI values versus the EI/respective shear stress (EI/SS) [36]. The slope of the best fit line provides the K_EI_ and the y-intercept corresponds to the EI_max_.

### Statistical analysis

Data are expressed as arithmetic means ± SEM. n denotes the number of different donor RBCs studied. Statistical analysis was performed using ANOVA with Tukey’s test as a post-test, t test or non-parametric Wilcoxon signed rank test by GraphPad Prism Version 8.4.3 (GraphPad Software, La Jolla, CA). A *P*-value less than 0.05 was considered statistically significant.

## Results

### Effect of thrombospondin-1 on Ca^2+^ homeostasis in human red blood cells

**Fig. 1:**
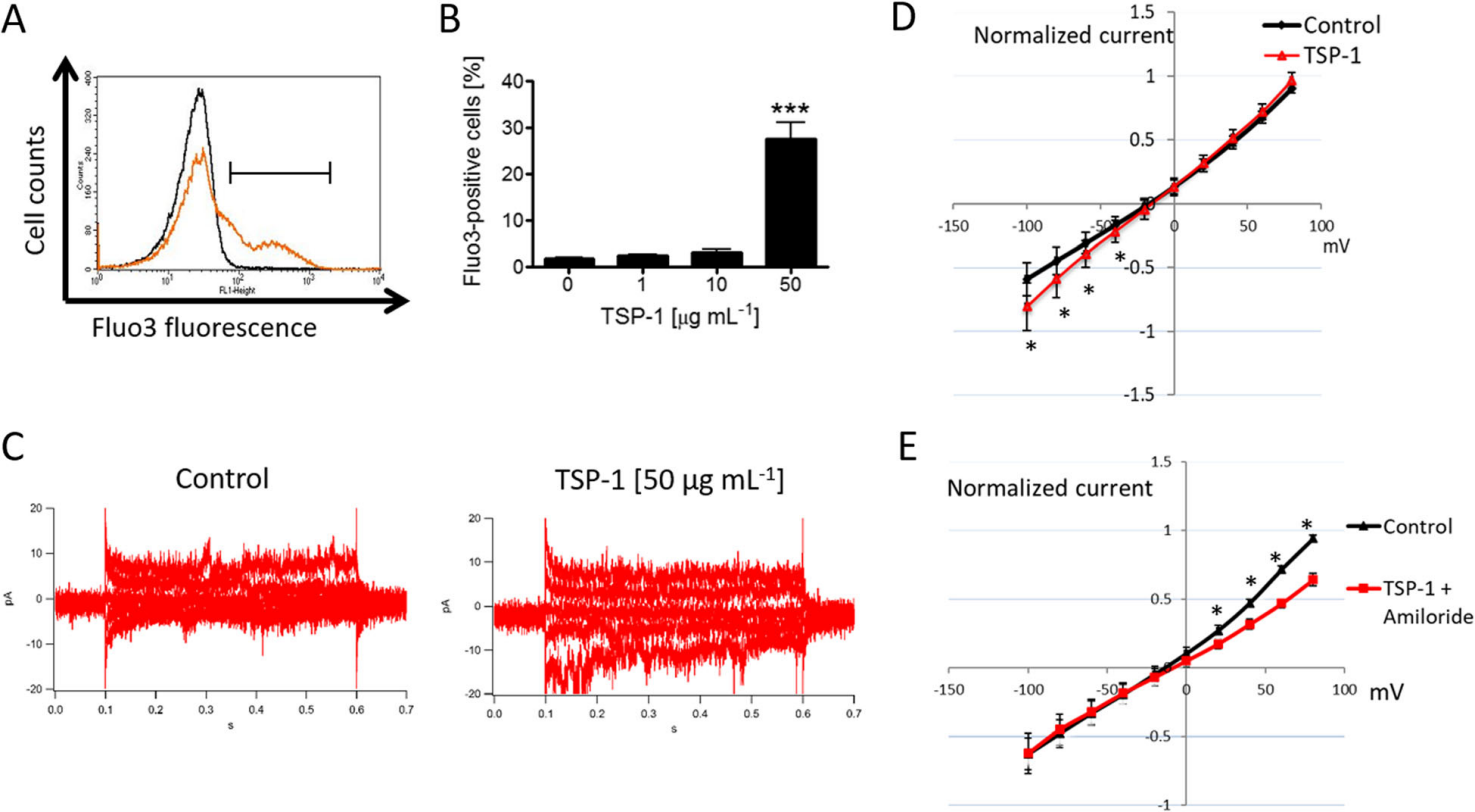
Effect of thrombospondin-1 on Ca^2+^ homeostasis in human red blood cells. Representative histogram (Black line: 0 μg/mL TSP-1, orange line: 50 μg/mL TSP-1; **a** and means ± SEM. **b** of Fluo3-positive RBCs (%) (*n* = 7) following a 48-h incubation at 37 °C in Ringer’s solution containing 0–50 μg mL^− 1^ TSP-1. *** indicate significant difference (*P* < 0.001) from the absence of TSP-1. **c** Raw current traces from a representative cell under control conditions (*left panel*) and in the presence of 50 μg/mL TSP-1 (*right panel*). For clarity, in both panels, not all traces, but every second one, starting with -100 mV, are being shown. Whole-cell currents were elicited by voltage steps from − 100 mV to 80 mV for 500 ms in 20 mV increments at 5 s intervals, Vh = − 30 mV. **d** I/V-curves in the absence (control; *black diamonds*) or in the presence of 50 μg/mL TSP-1 (*red triangles*; *n* = 11). Data are expressed as mean current ± SEM. * indicates significant difference (*P* < 0.05) from the absence of TSP-1. **e** I/V-curves in the absence (control; *black triangles*) or in the presence of 50 μg/mL TSP-1 and 1 mM amiloride (*red squares*; *n* = 11). Data are expressed as mean current ± SEM. * indicates significant difference (*P* < 0.05) from the absence of TSP-1 and amiloride

The impact of TSP-1 treatment on RBC intracellular Ca^2+^ levels was examined using Fluo3 fluorescence in flow cytometry analysis. As shown in Fig. 1a and b, exposure of RBCs to TSP-1 (50 μg/mL) for 48 h significantly enhanced the percentage of RBCs with increased Fluo3 fluorescence indicating increased cytoplasmic Ca^2+^ concentration. Whole-cell patch-clamp experiments were performed to elucidate whether TSP-1 influences cation channel activity. As illustrated in Fig. 1c and d, exposure of RBCs to 50 μg/mL TSP-1 using physiological internal and external solutions induced an increase in an inward conductance, indicating a possible cation flux into the cells, that may be related to the increase in the intracellular Ca^2+^ concentration. Furthermore, treatment with 1 mM amiloride, a cation channel inhibitor [28], abrogated the TSP-1-induced increase in the inward conductance (Fig. 1e). In addition, amiloride also blocked an outward current that was not induced by TSP-1.

### Effect of thrombospondin-1 on phosphatidylserine externalization, sphingomyelinase activation, and the generation of reactive oxygen species in human red blood cells

**Fig. 2:**
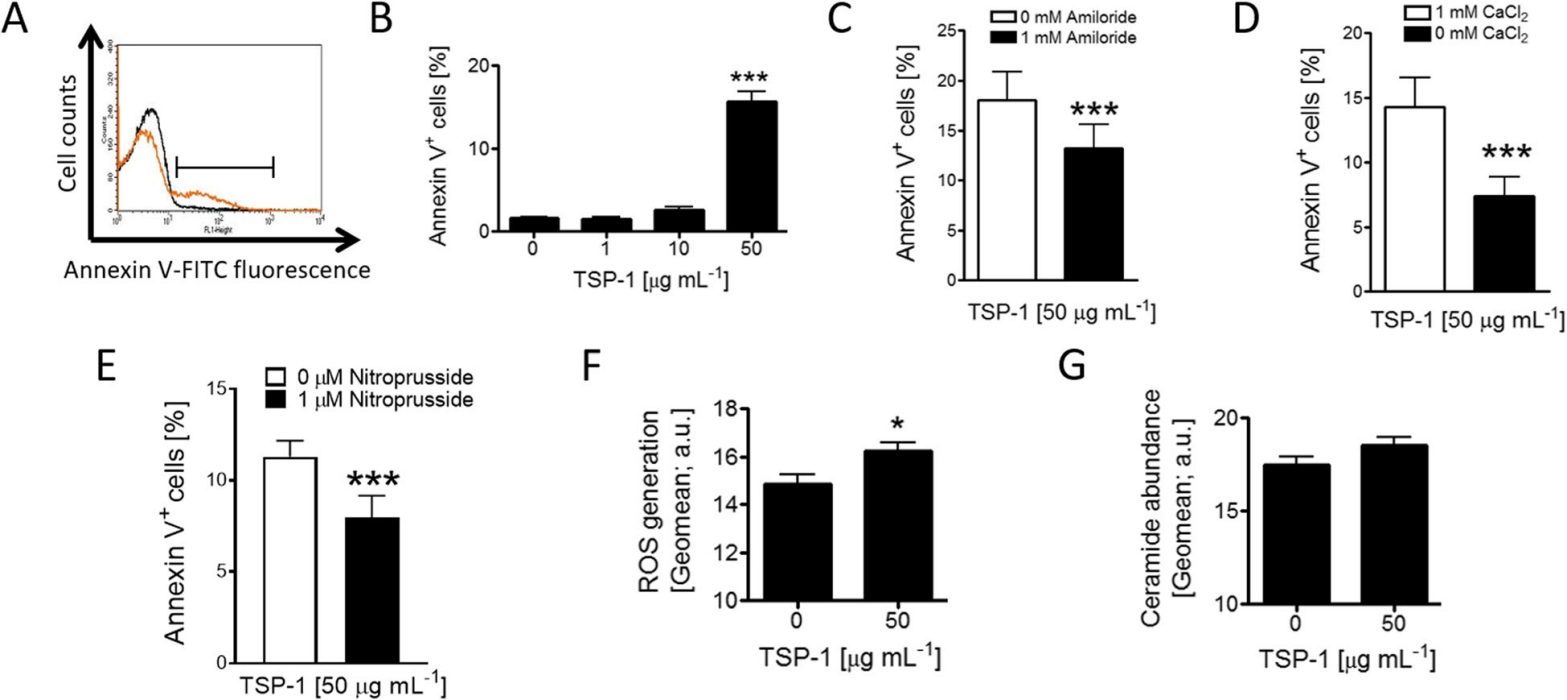
Effect of thrombospondin-1 on phosphatidylserine externalization, sphingomyelinase activation, and the generation of reactive oxygen species in human red blood cells. Representative histogram (Black line: 0 μg/mL TSP-1, orange line: 50 μg/mL TSP-1; **a** and means ± SEM. **b** of annexin V positive RBCs (*n* = 7) following 48-h incubation at 37 °C in Ringer’s solution containing 0–50 μg/mL TSP-1. *** indicate significant difference (*P* < 0.001) from the absence of TSP-1. Means ± SEM of annexin V positive RBCs following 48-h incubation in 50 μg/mL TSP-1 in the absence or presence of 1 mM amiloride (*n* = 27; **c**), 1 mM CaCl_2_ (*n* = 8; **d**) or 1 μM sodium nitroprusside (*n* = 12; **e**). *** indicates significant difference (*P* < 0.001) from the absence of amiloride, CaCl_2_ or sodium nitroprusside. Means ± SEM of the geometric means of DCFDA (*n* = 9; **f**) or ceramide-dependent (*n* = 14; **g**) fluorescence of RBCs following 48-h incubation without or with 50 μg/mL TSP-1. * indicates significant difference (*P* < 0.05) from the absence of TSP-1

Enhanced cytosolic Ca^2+^ content is expected to activate scramblases which, in turn, elicit cell membrane PS externalization. We observed that a 48-h incubation of RBCs in the presence of TSP-1 (50 μg/mL) significantly increased the percentage of annexin V-positive RBCs, reflecting cell membrane PS exposure (Fig. 2a and b). We then interrogated the extent to which Ca^2+^ influx contributes to PS externalization triggered by TSP-1. Blocking Ca^2+^ entry via NSCC using amiloride (1 mM [26]; Fig. 2c) or removal of extracellular Ca^2+^ (Fig. 2d) significantly blunted, but did not abolish, TSP-1-induced PS exposure suggesting that increased Ca^2+^ entry participates in, but does not completely account for, TSP-1-induced cell death. As TSP-1-induced PS exposure was not abolished by extracellular Ca^2+^ removal, we hypothesized that non-Ca^2+^-dependent mechanisms may contribute to the breakdown of phospholipid asymmetry. Multiple recent studies have shown the role of TSP-1 in modulating NO signaling in various cell types (reviewed in [3]). We, thus, explored whether NO-mediated signaling similarly modulates TSP-1-induced alterations in RBCs. As shown in Fig. 2e, treatment of RBCs with the NO donor sodium nitroprusside (1 μM) significantly reduced TSP-1-induced PS externalization, suggesting the involvement of this mechanism in concert with Ca^2+^-dependent signaling leading to RBC cell death. We then examined whether TSP-1 elicits oxidative stress and sphingomyelinase activation, putative RBC cell death effectors [21]. As shown in Fig. 2f and g, a 48-h exposure of RBCs to 50 μg/mL TSP-1 significantly enhanced DCFDA fluorescence, reflecting ROS production, but did not significantly enhance ceramide abundance suggesting that TSP-1 affects RBC redox balance favoring their suicidal death.

**Fig. 3:**
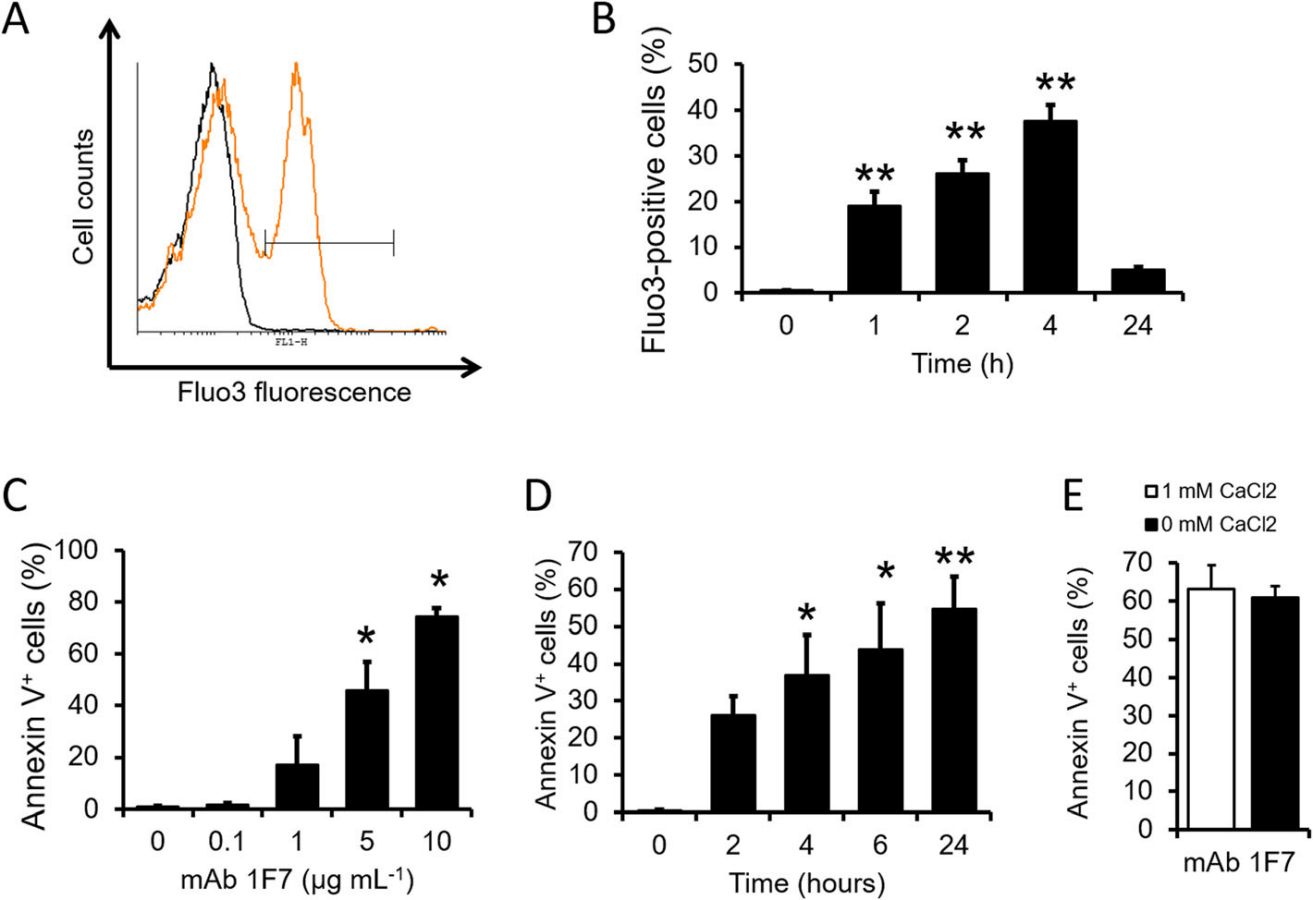
Effects of the anti-CD47 mAb 1F7 on cytosolic Ca^2+^ levels and phosphatidylserine exposure in human red blood cells. Representative histogram (Black line: 0 μg/mL mAb 1F7, orange line: 10 μg/mL mAb 1F7, following a 4-h incubation; **a**) and means ± SEM. **b** of Fluo3-positive RBCs (%) (*n* = 6; **b**) following incubation for 0–24 h at 37 °C in the presence of 10 μg/mL mAb 1F7. ** indicate significant difference (*P* < 0.01) from the zero time-point. **c** Means ± SEM of annexin V positive RBCs (*n* = 3) following 24-h incubation with 0–10 μg/mL mAb 1F7. * indicates significant difference (*P* < 0.05) from the absence of mAb 1F7. **d** Means ± SEM of annexin V positive RBCs (*n* = 5) following incubation with 10 μg/mL mAb 1F7 for 0–24 h. * and ** indicate significant difference (*P* < 0.05 and *P* < 0.01, respectively) from the zero time-point. **e** Means ± SEM of annexin V positive RBCs (*n* = 7) following 24-h incubation with 10 μg/mL mAb 1F7 in the absence or presence of 1 mM CaCl_2_

Since CD47 is a receptor for TSP-1 and CD47-signaling can regulate cytoplasmic Ca^2+^ dynamics [12], an anti-CD47 mAb could induce an increased RBC intracellular Ca^2+^ level. For this, anti-CD47 mAb 1F7 was used as it has been shown to induce apoptosis in other cell types [29, 31]. As illustrated in Fig. 3a and b, exposure of RBCs to mAb 1F7 (10 μg/mL) for 1–4 h significantly increased Fluo3 fluorescence. However, such an effect of mAb 1F7 was absent after a 24-h incubation (Fig. 3b). We observed that mAb 1F7 (0.1–10 μg/mL) dose-dependently increased the percentage of annexin V-positive RBCs after a 24-h incubation (Fig. 3c). In addition, there was a time-dependent increase in the percentage of annexin V-positive RBCs in response to mAb 1F7 (Fig. 3d). Interestingly, similar levels of RBC PS exposure in response to mAb 1F7 were also seen in the absence of extracellular Ca^2+^ during incubation (Fig. 3e).

### Effect of thrombospondin-1 on morphology and deformability of human red blood cells

**Fig. 4:**
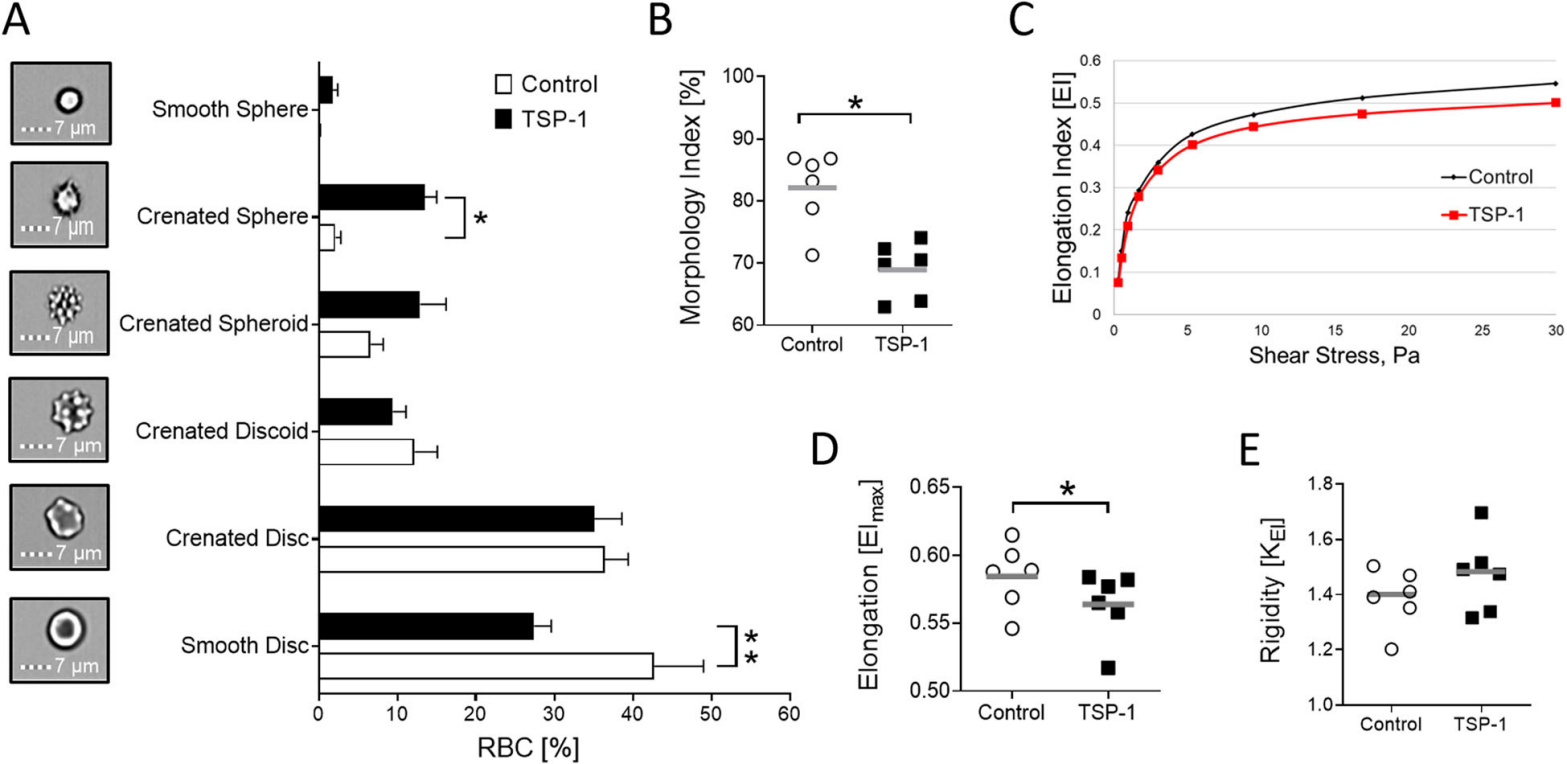
Effect of thrombospondin-1 on morphology and deformability of human red blood cells. Means ± SEM showing distribution of RBC morphology (*n* = 6; **a**) following a 48-h incubation at 37 °C in Ringer’s solution in the absence (Control) or presence of TSP-1 (50 μg/mL). RBC morphology was assessed using Bright Field images from ImageStream X MkII (60x magnification). Morphology index (*n* = 6; **b**) following a 48-h incubation of RBCs at 37 °C in the absence (Control) or presence of TSP-1 (50 μg/mL). **c** Representative deformability curve (for RBCs from a single donor) of untreated (Control; black line) and TSP-1-treated (red line) RBCs obtained from LORRCA prior to Eadie-Hofstee linearization. Maximum elongation index (EI_max_; *n* = 6; **d**) and rigidity (K_EI_; *n* = 6; **e**) following a 48-h incubation of RBCs in the absence (Control) or presence of TSP-1 (50 μg/mL). * and ** indicate significant difference (*P* < 0.05 and *P* < 0.01, respectively) from the absence of TSP-1. Gray lines indicate means

The effect of TSP-1 on RBC morphology and deformability was determined. In imaging flow cytometry analyses, it was observed that the proportion of RBCs with smooth disc shape was significantly reduced and RBCs with crenated sphere shape were significantly increased after a 48-h incubation in the presence of TSP-1 (Fig. 4a). Accordingly, as shown in Fig. 4b, TSP-1 treatment significantly reduced the morphology index of RBCs. Ektacytometry analyses revealed that TSP-1 treatment affected RBC deformability (Fig. 4c). As depicted in Fig. 4d, in comparison to untreated RBCs, TSP-1 (50 μg/mL) exposure for 48 h significantly reduced maximum elongation index (EI_max_) suggesting that TSP-1 induces increased RBC stiffness. Furthermore, TSP-1 (50 μg/mL) treatment tended to increase K_EI_ reflecting RBC rigidity (Fig. 4e). Thus, the ability of RBCs to adopt a new shape in response to deforming forces, which dictate their rheological properties, is affected by TSP-1.

### Effect of thrombospondin-1 on microvesiculation of human red blood cells

**Fig. 5:**
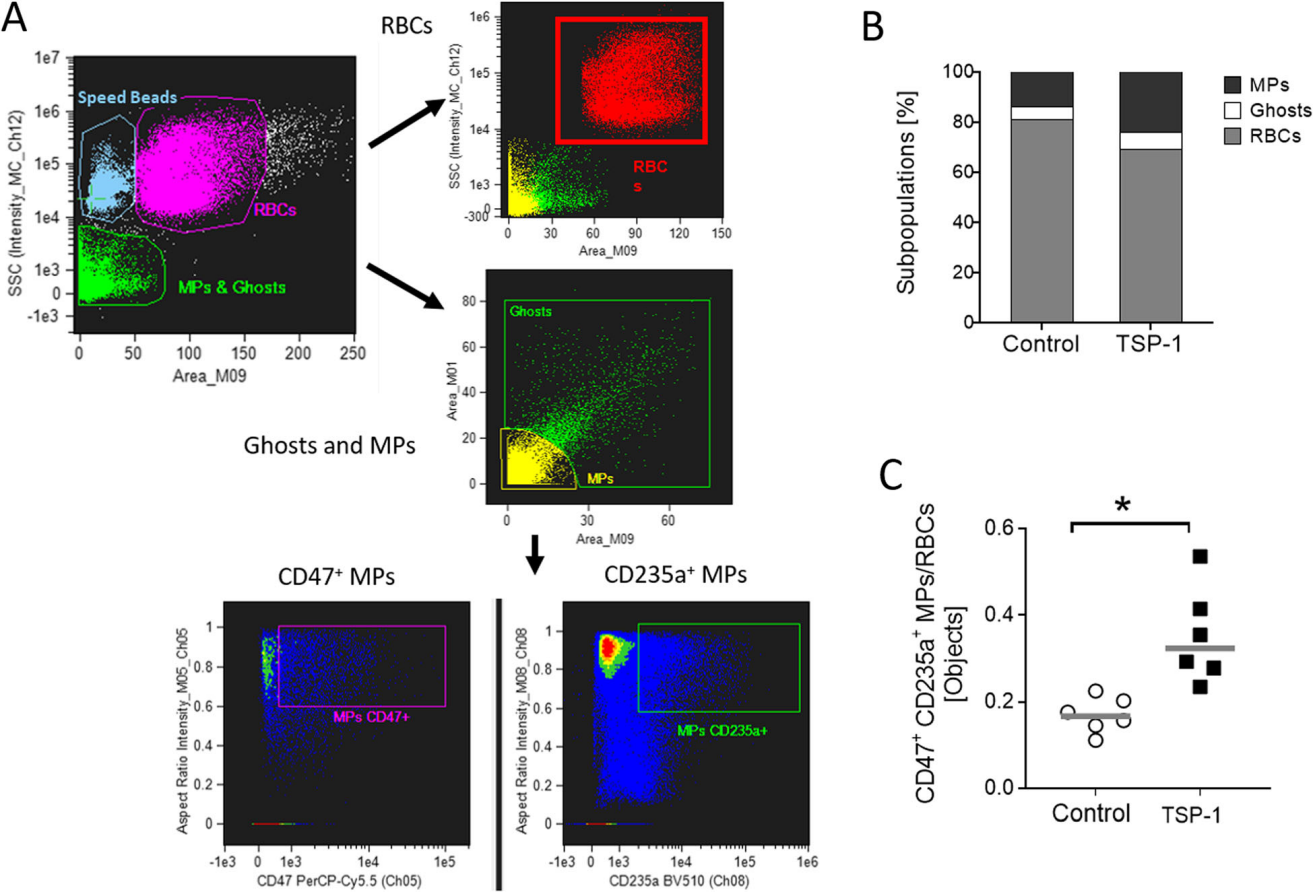
Effect of thrombospondin-1 on microvesiculation of human red blood cells. Gating strategy in imaging flow cytometry analysis (**a**). The Dark Field (DF) Scattering intensity (SSC, Ch12) Area and Aspect Ratio Features (including two Bright Fields (BF) (BF1, Ch01 or BF2, Ch09) were used to differentiate RBC, ghost, and MP subpopulations. Percentages of RBC (gray), ghost (white), and MPs (black) in RBC samples incubated for 48 h in Ringer’s solution at 37 °C (**b**). Ratio of CD47^+^ CD235a^+^ MPs to RBCs after 48-h incubation at 37 °C in the absence (Control) or presence of TSP-1 in Ringer’s solution (*n* = 6; **c**). Gray lines indicate means. *indicates significant difference (*P* < 0.05) from the absence of TSP-1

The impact of TSP-1-induced RBC dysfunction on microvesiculation was assessed. As shown in Fig. 5b and c, incubation of RBCs with TSP-1 for 48 h significantly increased CD47^+^/CD235a^+^ MPs relative to RBC count, as compared to untreated RBCs indicating that TSP-1 promotes MP shedding.

## Discussion

Compelling molecular evidence points to an essential role for CD47-dependent TSP-1 signaling in the pathophysiology of a wide range of systemic diseases [3–6]. However, little is known about this signaling mechanism in influencing anucleate RBC functions. Increase of cytoplasmic Ca^2+^ levels is a vital element in potentiating premature cell death and clearance of circulating RBCs [21, 37]. In the current study, we demonstrate, for the first time, that exogenous TSP-1 causes RBC dysfunction evoking an increase in intracellular Ca^2+^ levels, triggering cell death, and altering cell morphology and rheological properties.

Increased intracellular Ca^2+^ concentration in RBCs, triggered by the opening of NSCC, stimulates phospholipid scrambling, bleb formation, and vesiculation of the cell membrane [21, 28]. Enhanced cytosolic Ca^2+^ is further involved in the activation of multiple Ca^2+^-sensitive enzymes such as transglutaminases, phospholipases, calpains, protein kinases and phosphatases [21]. While the molecular identity of the cation channels remains incompletely characterized, it is believed to involve the TRPC6 channel [21, 28]. According to our data, the TSP-1-elicited increase in the cytosolic Ca^2+^ concentration could be corroborated using whole-cell patch-clamp recordings which showed the presence of a TSP-1-induced inward current, alluding to a possible Ca^2+^ influx. In addition, we also observed that ligation of the anti-CD47 mAb 1F7 induced an increase in RBC cytosolic Ca^2+^ levels. These findings are consistent with previous studies in nucleated cells, which suggest that CD47/TSP-1 signal transduction impacts cellular Ca^2+^ homeostasis [38–40]. Notably, intact TSP-1 was previously demonstrated to upregulate intracellular Ca^2+^ levels in fibroblasts; this effect was recapitulated by the TSP-1-derived peptide RFYVVMWK underlining the primordial role of TSP-1/CD47 signaling in regulating cytoplasmic Ca^2+^ levels [38]. Furthermore, cardiac myocytes treated with 7 N3, a peptide derived from the C-terminal of TSP-1, displayed acutely elevated intracellular Ca^2+^ levels through the release of Ca^2+^ from the sarcoplasmic reticulum [39].

Ample evidence underscores the role of oxidative stress in modulating RBC Ca^2+^ homeostasis and survival by stimulating NSCC conductance [41]. In accordance, our data reveal that TSP-1 treatment stimulated a subtle increase in RBC ROS production, which, in turn, may favor Ca^2+^ entry and promote cell death. TSP-1 has previously been shown to potentiate ROS generation in vascular smooth muscle cells via CD47-dependent activation of NADPH oxidase 1 [42]. TSP-1 has further been implicated in oxidative stress-mediated renal ischemia-reperfusion injury by stimulating ROS production in renal tubular endothelial cells [43]. In addition, the present study also revealed that pharmacological NO supplementation significantly blunted TSP-1-induced PS externalization in RBCs. NO was previously shown to influence RBC survival by modulating cell death pathways downstream of intracellular Ca^2+^ increase, but not by directly influencing Ca^2+^ entry *per se* [44]. In purview of these findings, NO has previously been documented to be an essential effector of TSP-1 signaling in a wide range of cell types, and is associated with various clinical conditions [3, 45].

RBC CD47 serves as a putative molecular switch in erythrophagocytosis [46]. Through activation of signaling mediated by tyrosine phosphatases, downstream of its interaction with SIRPα, CD47 inhibits phagocytosis, and thereby functions as a “do not eat me” signal [11]. Paradoxically, however, CD47 in experimentally aged RBCs was shown to undergo a conformational change and increased binding to TSP-1, which, in turn, promoted phagocytosis [46]. It is, therefore, possible that PS externalization during RBC cell death induced by TSP-1/CD47 signaling contributes, at least in part, to this “eat me” response.

RBCs exhibit an extraordinary ability to deform which facilitates their smooth passage in the microcirculation and, thus, aids in maintaining optimal rheology [47]. Increased RBC stiffness facilitates the elimination of senescent and injured RBCs from the circulation in the spleen [48]. Previous studies have elucidated the pivotal role of RBC NO synthase-derived NO in the regulation of RBC deformability [49, 50]. On the other hand, elevated cytoplasmic Ca^2+^ levels in RBCs are associated with reduced deformability [51]. Along these lines, we observed that TSP-1 treatment altered the indices of RBC deformability at exposure durations, which also elicited both enhanced cellular Ca^2+^ concentration and cell death. As RBC rigidity is an important hemorheological parameter leading to reduced blood viscosity, our findings may explain the occurrence of vaso-occlusive events associated with enhanced TSP-1 plasma levels [52].

TSP-1 has previously been implicated in the pathophysiology of vascular occlusion and pulmonary hypertension associated with sickle cell disease (SCD) [53, 54]. Increased prothrombotic risk in SCD is linked to elevated TSP-1 levels, which not only inhibit ADAMTS13 proteolysis of von Willebrand Factor [55], but also provoke RBC MP shedding; this process, in turn, favors RBC adhesion to endothelial cells as well as stimulation of endothelial cell apoptosis [56]. Our data confirm MP shedding from RBCs following exposure to TSP-1 in vitro. Mechanistically, MP shedding by RBCs, as elucidated during their storage under blood banking conditions, may be a consequence of ATP depletion, K^+^ leakage, and elevation of intracellular Ca^2+^ [16] It may, therefore, be inferred that TSP-1-induced increase of intracellular Ca^2+^ concentration leads to activation of Ca^2+^-dependent proteases leading to cytoskeletal damage and MP shedding [16]. Intriguingly, both TSP-1 and the CD47 agonist 4 N1–1 have been shown to potentiate the transformation of cell shape in SCD from discocytes to echinocytes [56]. It is well established that an increased proportion of RBCs in SCD patients expose procoagulant PS on their surface, which may lead to thrombosis [57]. It is, therefore, reasonable to conjecture that hyperactive cation currents in RBCs are an important underlying mechanism of RBC dysfunction and thrombosis in SCD patients. TSP-1 may contribute to this channel activation.

Accelerated cell death of RBCs has been shown to occur in a variety of systemic conditions and may contribute to anemia, thrombosis, and impaired microcirculation in these disorders [21]. At least in theory, increased TSP-1 levels, encountered in these conditions, may aggravate RBC Ca^2+^ entry leading to RBC cell death [21]. Remarkably, TSP-1 serum concentrations were documented to be 100-fold higher than plasma concentrations indicating TSP-1 release by platelets [58]. TSP-1 concentrations used in this study (1–50 μg/mL) are well in the range of plasma and serum levels achieved in conditions such as SCD [53], and interstitial pneumonia [59], respectively. Furthermore, 100 μg/mL of TSP-1 was previously used to demonstrate the impact of CD47 ligation on RBC viability in vitro [20].

## Conclusions

Taken together, our data unravel that TSP-1/CD47 signaling mediates enhanced RBC Ca^2+^ concentration contributing to cell death. Targeting this signaling pathway may represent a possible therapeutic option in mitigating RBC-related pathophysiology in different clinical conditions associated with elevated TSP-1 levels.

## Data Availability

The datasets used and/or analyzed during the current study are available from the corresponding author on reasonable request.

## Abbreviations

ATP: Adenosine triphosphate
DCFDA: 2′,7′-dichlorodihydrofluorescein diacetate
EI_max_: Maximum elongation index
K_EI_: Rigidity
LORRCA: Laser-assisted optical rotational red cell analyzer
MP: Microparticles
NO: Nitric oxide
NSCC: Non-selective cation channels
PS: Phosphatidylserine
PVP: Polyvinylpyrrolidone
RBC: Red blood cell
ROS: Reactive oxygen species
SCD: Sickle cell disease
SIRPα: Signal regulatory protein α
TSP-1: Thrombospondin 1
